# Validation and application of a novel in vivo cervical spine kinematics analysis technique

**DOI:** 10.1038/s41598-021-01319-x

**Published:** 2021-12-20

**Authors:** Zongmiao Wan, Wenjin Wang, Chao Li, Junjie Li, Jinpeng Lin, Fei Tian, Ting Zhu, Danni Wu, Luqi Guo, Shaobai Wang

**Affiliations:** 1 Department of Orthopedics, The First Affiliated Hospital of Nanchang University, Nanchang, 330000 Jiangxi China; 2 Key Laboratory of Exercise and Health Sciences of Ministry of Education, School of Kinesiology, Shanghai University of Sport, Shanghai, 20043 China; 3 Institute of Biomechanics and Orthopaedics, German Sport University Cologne, 50933 Cologne, Germany; 4 Department of Rehabilitation Medicine, Heping Hospital Affiliated To Changzhi Medical College, Shanxi, 046000 China; 5 Present Address: School of Kinesiology, Shanghai University of Sport, Research Building 412, 200 Hengren Road, Shanghai, 200438 China

**Keywords:** Health care, Medical research

## Abstract

To validate the accuracy of Cone beam computed tomography (CBCT) cervical spine modeling with three dimensional (3D)-3D registration for in vivo measurements of cervical spine kinematics. CBCT model accuracy was validated by superimposition with computed tomography (CT) models in 10 healthy young adults, and then cervical vertebrae were registered in six end positions of functional movements, versus a neutral position, in 5 healthy young adults. Registration errors and six degrees of freedom (6-DOF) kinematics were calculated and reported. Relative to CT models, mean deviations of the CBCT models were < 0.6 mm. Mean registration errors between end positions and the reference neutral position were < 0.7 mm. During flexion–extension (F–E), the translation in the three directions was small, mostly < 1 mm, with coupled LB and AR both < 1°. During lateral bending (LB), the bending was distributed roughly evenly, with coupled axial rotation (AR) opposite to the LB at C1–C2, and minimal coupled F–E. During AR, most of the rotation occurred in the C1–C2 segment (29.93 ± 7.19° in left twist and 31.38 ± 8.49° in right twist) and coupled LB was observed in the direction opposite to that of the AR. Model matching demonstrated submillimeter accuracy in cervical spine kinematics data. The presently evaluated low-radiation-dose CBCT technique can be used to measure 3D spine kinematics in vivo across functional F–E, AR, and LB positions, which has been especially challenging for the upper cervical spine.

## Introduction

The lifestyle changes that come with nation development have been associated with increased incidences and earlier onset of cervical spine pathology^1–3^. Accurate measurement of spine kinematics is helpful for characterizing intervertebral changes, investigating biomechanical mechanisms of cervical spine pathology, and evaluating surgical outcomes^4–9^, including assessing the effects of vertebral fusion on the mobility of adjacent vertebrae^10^.

Traditionally, kinematic analyses of the cervical spine have been based on cadaveric simulations and on two- and three-dimensional (2D and 3D) images. Cadaveric studies cannot reproduce joint motion accurately due to the lack of muscular activities and a physiological environment^11^. The main limitation of 2D images, such as radiographs^10,11^, is lack of information about 3D movement characteristics^14,15^. Although computed tomography (CT) and magnetic resonance imaging (MRI) techniques can be used to obtain 3D in vivo measurements^16,17^, they are performed in subjects that are lying down and thus without physiological loading. Additionally, MRI, CT, and fluoroscopy have the respective drawbacks of a long scanning time, substantial radiation exposure, and motion artifacts. Cone beam computed tomography (CBCT) is a state-of-the-art imaging technique that provides diagnostic quality images with reasonably low radiation dose scanning protocols. The generation of 3D reconstructions of cervical spine structures were reported in several studies^18–20^.

Quantitative knowledge of in vivo cervical spine kinematics is important for understanding cervical spine pathology and for improving surgical treatment of cervical spine degenerative disease. The purpose of the present study was to validate the accuracy of CBCT modeling and a 3D–3D registration technique for the measurement of in vivo cervical spine kinematics. We hypothesize that the technique can be used for segmental kinematic analysis of the cervical spines, especially at the end positions of functional movements.

## Methods

The validation and application of 3D–3D registration technique for in vivo measurement of cervical spine kinematics was conducted in three phases. In the first phase, we validated our CBCT models’ accuracy by superimposing them with corresponding CT models. In the second phase, cervical vertebrae were registered in six end positions of the movement relative to a corresponding neutral position. In the third phase, we applied this method to obtain measurements over six degrees of freedom (6-DOF) of both the upper and subaxial cervical spine vertebrae.

### Participants

A group of 10 healthy young adults (5 women and 5 men) with a mean age (± standard deviation) of 30.20 ± 6.11 years participated in the first phase of the study. Subsequently, 5 healthy young adults (3 women and 2 men) with a mean age of 27.8 ± 6.7 years, who did not participate in phase 1, participated in the second and third phases. All participants signed an informed consent form prior to participation, and identifying information/images have been obtained informed consent. The study protocol was approved by the institutional review board at the First Affiliated Hospital of Nanchang University (2020, no. 46), and all methods were performed in accordance with the relevant guidelines and regulations. Radiation safety approval was obtained.

### Data acquisition

In the first phase, each participant received a CT (SOMATOM Definition AS +) and a CBCT (I-CAT, KaVo3DeXam, American) scan. The CT parameters were: slice thickness, 0.6 mm; pixel size, 0.25 mm; tube current, 190 mA; 130 kV; and scan time, 8.6 s. The CBCT parameters were: slice thickness, 0.2 mm; pixel size, 0.25 mm; tube current, 20.27 mA, voltage, 120 kV; and rotation (scan) time 14.7 s. The CT and CBCT datasets were used to construct surface models of each subject’s vertebrae via segmentation and reconstruction processed using solid modeling software (Amira 6.7.0 Thermo Fisher Scientific, Rockford, IL).

**Figure 1:**
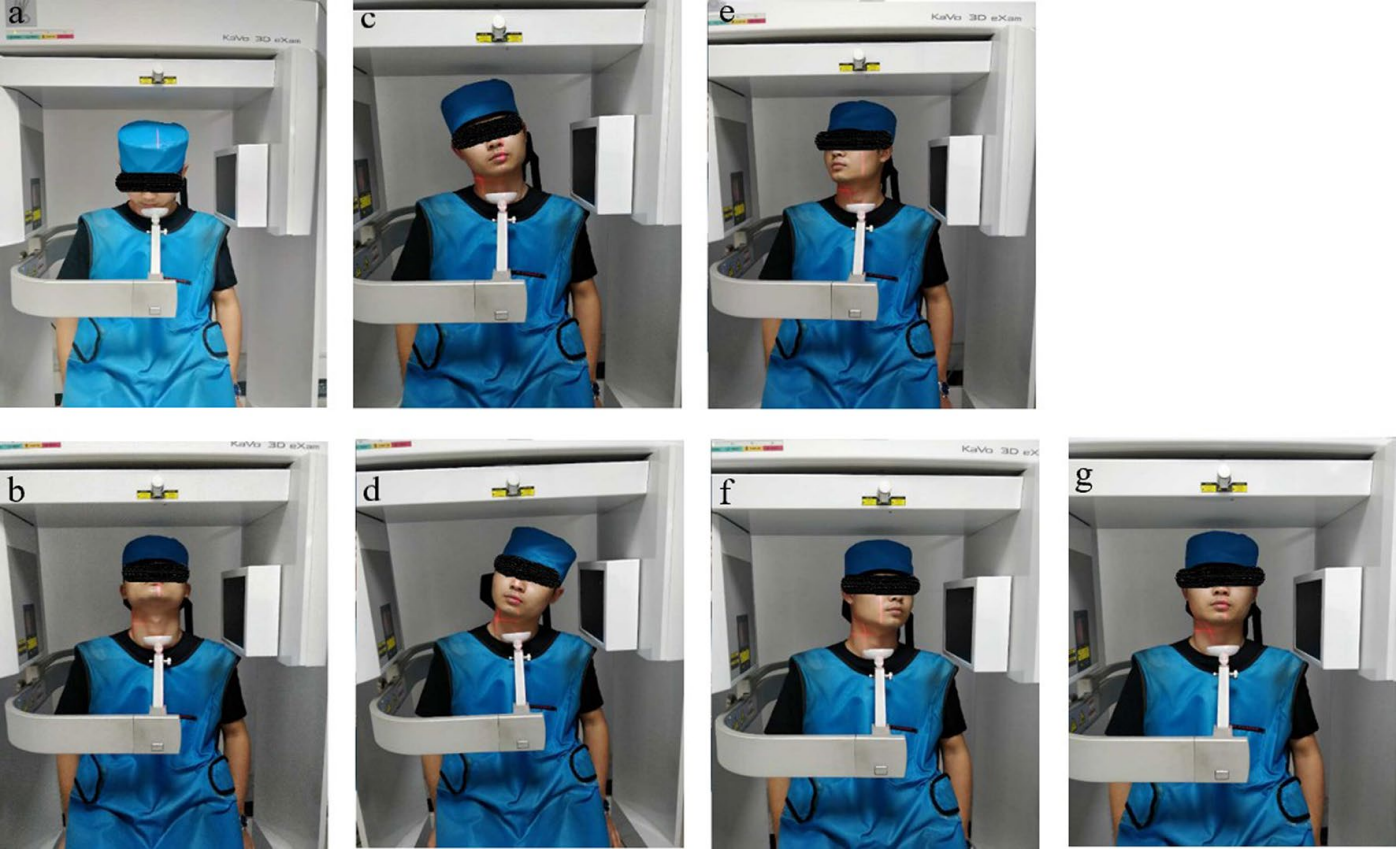
Overview of cone-beam computed tomography (CBCT) scanning showing a participant performing (**a**,**b**) flexion–extension (F–E), (**c**,**d**) lateral bending (LB), and (**e**,**f**) axial rotation (AR) movements, as well as (**g**) a neutral position.

**Figure 2:**
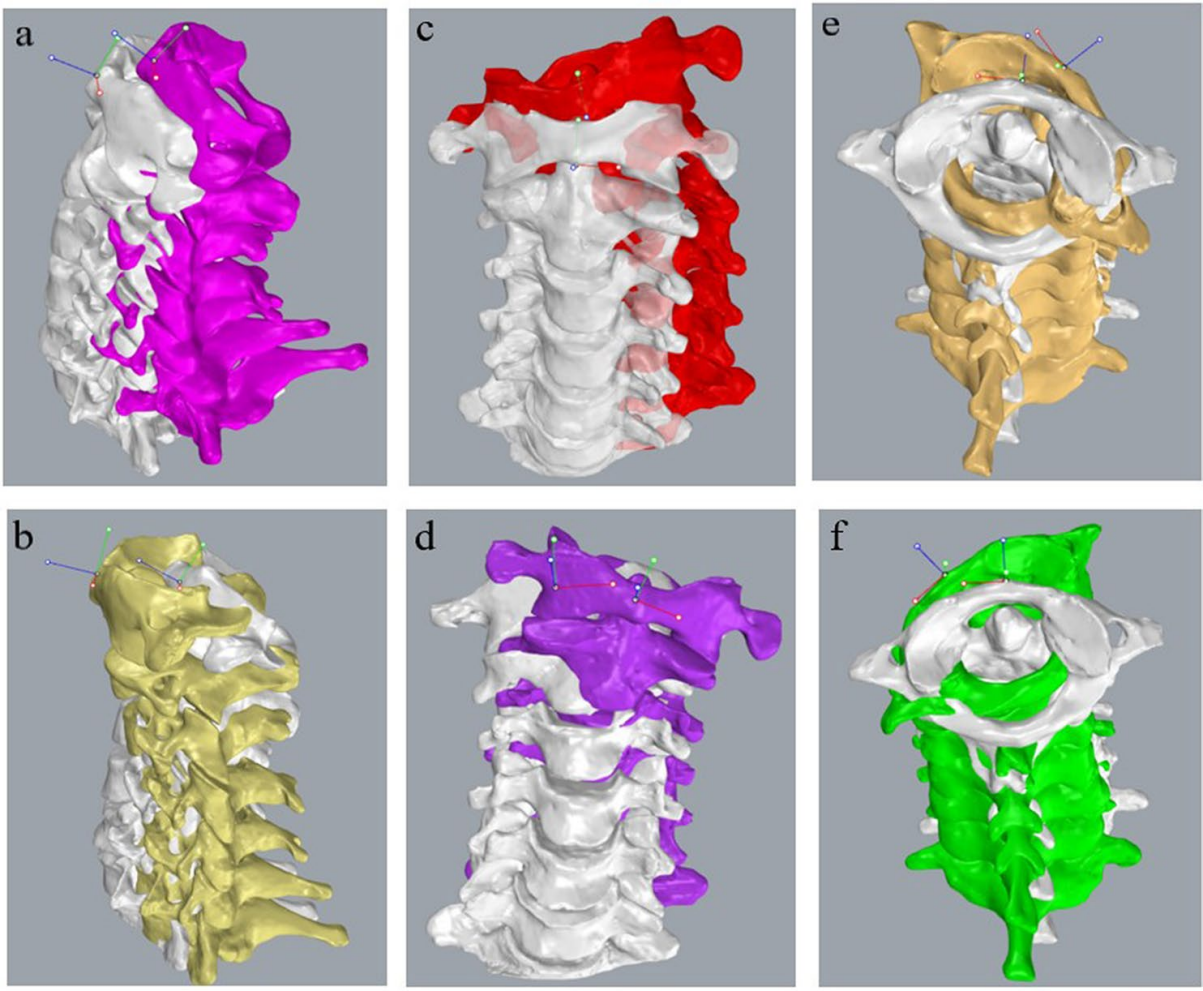
Three-dimensional (3D) models of the cervical spine of a representative healthy young male subject. (**a**,**b**) F-E models. (**c**,**d**) LB models. (**e**,**f**) AR models. The functional range-of-motion positions are pseudo-colored and shown together with a grey 3D model representing the neutral position.

In the second and third phases, each participant received only CBCT scans. The CBCT datasets were used to construct surface models of each subject’s vertebrae via segmentation and reconstruction processed using solid modeling software (Amira). The participants performed active functional head flexion–extension (F–E), functional lateral bending (LB) in which the head was bent down leftward and then rightward, functional axial rotation (AR) in which the head was rotated to the left and the right, and neutral tasks while sitting on a chair with a stabilized trunk, wearing a lead apron to protect the lower body from radiation (Figs. 1 and 2). Data for all seven positions were collected in a single trial with a total radiation dosage of ~ 68.7 µSv (≈2% of a standard neck CT, which is 3 mSv).

Before data collection, each participant was asked to practice all of the movements several times under the guidance of an experienced instructor until s/he was able to perform the motions without any guidance. During head F–E, participants bent their heads first forward and then backward to maximum stretch positions without discomfort. During head LB, they bent their heads downward toward the left shoulder and then downward toward the right shoulder to maximal positions without discomfort. During head AR, they rotated their heads first leftward and then rightward to maximal rotation positions without discomfort. After each stretch, the participants returned their heads to a neutral position. The patients took CT scans in conventional supine position. They took CBCT scans in sitting position.

### Model registration

In the first phase, automated registration technology (ART) was used to superimpose each participant’s CT segmental model onto its corresponding CBCT segmental model in the neutral position (Fig. 1) using Fluo-Motion software (v1.0, Innomotion Inc., Shanghai, China). In the second phase, ART was used to superimpose the neutral segmental model onto each corresponding end position of the movement segmental models in Fluo-Motion software. A 3D-3D cervical segmental model registration was performed based on iterative closest point method^21^. Each moving cervical segmental model was translated and rotated iteratively in space to align its corresponding target model in the neutral position. The summation of perpendicular distances of moving model vertices was calculated with respect to the closest target model surfaces. Optimization alignment was obtained when the summation reached minimum.

### Kinematic analysis

**Figure 3:**
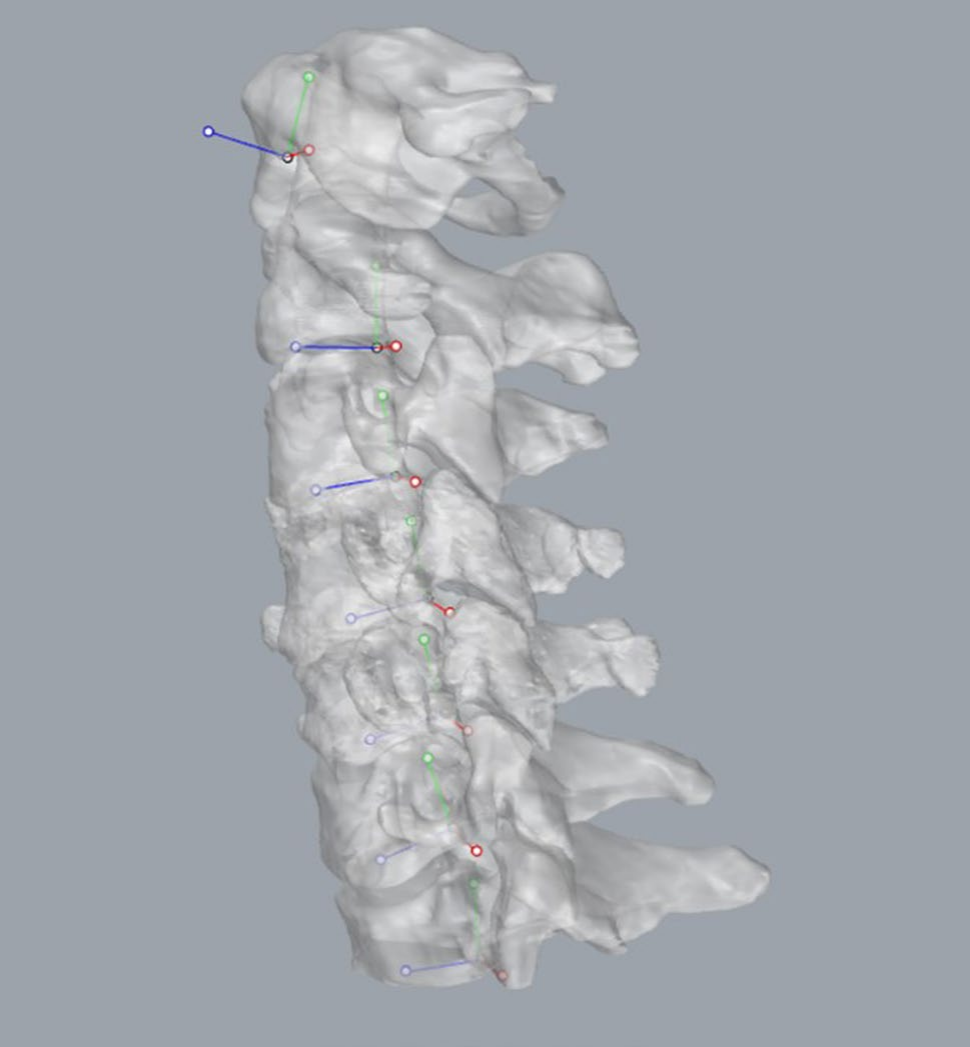
Schematic of the anatomic coordinate system employed in this study. Red arrows show F-E axes, blue arrows show LB axes, and green arrows show AR axes.

To describe 3D segmental motion characteristics, each model was embedded in an anatomic coordinate system defined by vertebra features^22^ (Fig. 3). The coordinate system originated at the most posterior-inferior point of the vertebral body in the mid-sagittal plane, with a left-going positive x axis, the positive y axis directed superiorly, and the positive z axis directed anteriorly. Intervertebral joint angles were determined based on the rotation of the coordinate system of each vertebra relative to that of the subjacent vertebra. Intervertebral translations were similarly defined as the relative displacement of the origins. We used mm for translation and deg (°) for rotation.

## Results

**Figure 4:**
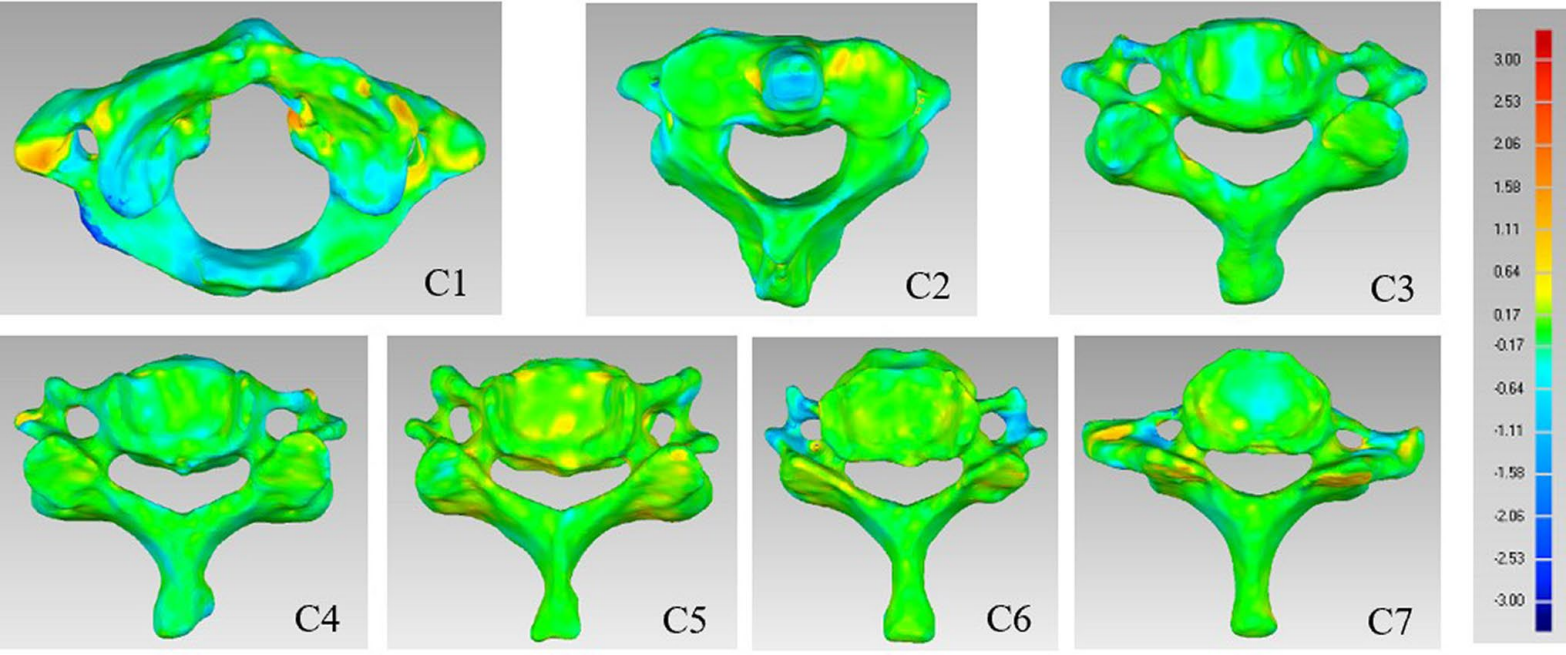
Superimposition of CBCT models with corresponding CT models of each cervical vertebra in the neutral position. Deviations between the CBCT and CT models are indicated with the color bar. Units, mm.

Mean deviations of CBCT models from their corresponding superimposed CT models (range 0.30–0.57 mm) are reported in Table 1. The mean deviation ranges of the six end positions of functional movements from the neutral position for the CBCT model were 0.14–0.67 mm during head F–E (Table 2), 0.15–0.66 mm during LB (Table 3), and 0.14–0.65 mm during head AR (Table 4). The local fit levels of the models are indicated with a color-bar in Fig. 4. Usually, maximum deviations occurred at the tip of the transverse process or bony structure edges, which should have little influence on overall kinematic analyses.

**Table 1:** Accuracy of CBCT models of cervical spine vertebrae relative to CT models.

Level	Average distance				Maximum distance		Standard deviation	Angular prediction
	Mean deviation	Mean absolute deviation	+	−	+	−		
C1	0.03	0.30	0.33	− 0.27	3.42	− 3.52	0.41	0.14
C2	0.02	0.24	0.25	− 0.22	2.23	− 2.46	0.30	0.10
C3	0.06	0.24	0.29	− 0.18	2.31	− 2.05	0.32	0.30
C4	0.06	0.25	0.30	− 0.19	2.28	− 2.36	0.35	0.30
C5	0.06	0.26	0.32	− 0.20	2.48	− 2.46	0.36	0.30
C6	0.04	0.29	0.32	− 0.25	2.70	− 2.74	0.39	0.20
C7	0.05	0.39	0.34	− 0.44	3.13	− 3.20	0.57	0.24

Units, mm, °.

**Table 2:** Accuracy of CBCT-modeled cervical spine vertebrae across functional E–F position models relative to a neutral position model.

Posture	Level	Average distance				Maximum distance		Standard deviation	Angular prediction
		Mean deviation	Mean absolute deviation	+	−	+	−		
Flexion	C1	0.02	0.21	0.22	− 0.19	3.20	− 2.90	0.31	0.10
	C2	0.01	0.18	0.17	− 0.19	2.23	− 2.63	0.24	0.05
	C3	0.02	0.18	0.16	− 0.20	1.84	− 2.08	0.22	0.10
	C4	0.02	0.21	0.19	− 0.23	1.96	− 2.12	0.24	0.10
	C5	0.03	0.18	0.16	− 0.22	1.38	− 2.31	0.22	0.14
	C6	0.00	0.19	0.19	− 0.19	1.92	− 2.56	0.25	0.00
	C7	0.04	0.38	0.42	− 0.34	3.62	− 3.39	0.67	0.20
Extension	C1	0.01	0.19	0.18	− 0.20	2.85	− 3.35	0.31	0.05
	C2	0.00	0.13	0.13	− 0.13	1.90	− 2.19	0.19	0.00
	C3	0.02	0.09	0.11	− 0.07	1.76	− 2.10	0.16	0.10
	C4	0.02	0.09	0.11	− 0.07	1.34	− 1.32	0.14	0.10
	C5	0.02	0.11	0.12	− 0.09	1.97	− 1.56	0.15	0.10
	C6	0.01	0.12	0.12	− 0.11	2.48	− 2.50	0.18	0.05
	C7	0.02	0.21	0.22	− 0.19	3.20	− 2.90	0.31	0.10

Units, mm, °.

**Table 3:** Accuracy of CBCT-modeled cervical spine vertebrae across functional LB position models relative to a neutral position model.

Posture	Level	Average distance				Maximum distance		Standard deviation	Angular prediction
		Mean deviation	Mean absolute deviation	+	−	+	−		
Right bend	C1	0.00	0.19	0.19	− 0.19	3.22	− 2.37	0.33	0.00
	C2	0.00	0.14	0.14	− 0.14	2.07	− 2.56	0.23	0.00
	C3	0.00	0.14	0.14	− 0.14	1.96	− 2.23	0.20	0.00
	C4	0.02	0.14	0.12	− 0.15	1.90	− 2.47	0.17	0.10
	C5	0.02	0.12	0.10	− 0.13	1.80	− 2.02	0.15	0.10
	C6	0.00	0.11	0.11	− 0.11	1.46	− 2.34	0.16	0.00
	C7	0.03	0.40	0.43	− 0.37	3.29	− 3.43	0.64	0.14
Left bend	C1	0.01	0.19	0.18	− 0.19	2.30	− 2.77	0.28	0.05
	C2	0.01	0.16	0.15	− 0.16	2.11	− 2.43	0.22	0.05
	C3	0.02	0.17	0.18	− 0.15	2.04	− 2.21	0.24	0.10
	C4	0.01	0.13	0.12	− 0.14	1.87	− 2.29	0.18	0.05
	C5	0.01	0.14	0.13	− 0.14	2.14	− 2.30	0.18	0.05
	C6	0.01	0.13	0.13	− 0.12	1.44	− 1.72	0.18	0.05
	C7	0.01	0.44	0.45	− 0.43	3.20	− 3.34	0.66	0.05

Units, mm, °.

**Table 4:** Accuracy of CBCT-modeled cervical spine vertebrae across functional AR position models relative to a neutral position model.

Posture	Level	Average distance				Maximum distance		Standard deviation	Angular prediction
		Mean deviation	Mean absolute deviation	+	−	+	−		
Right rotation	C1	0.00	0.19	0.20	− 0.20	2.41	− 2.49	0.29	0.00
	C2	0.00	0.14	0.15	− 0.14	2.32	− 1.82	0.21	0.00
	C3	0.00	0.14	0.13	− 0.14	1.75	− 1.86	0.18	0.00
	C4	0.02	0.14	0.10	− 0.11	1.71	− 1.28	0.14	0.10
	C5	0.02	0.12	0.11	− 0.11	1.76	− 1.76	0.15	0.10
	C6	0.00	0.11	0.12	− 0.12	1.58	− 2.32	0.18	0.00
	C7	0.03	0.40	0.29	− 0.35	3.07	− 3.59	0.52	0.14
Left rotation	C1	0.01	0.19	0.18	− 0.19	2.25	− 2.46	0.27	0.05
	C2	0.00	0.15	0.15	− 0.15	2.19	− 1.88	0.21	0.00
	C3	0.00	0.13	0.13	− 0.13	1.58	− 1.95	0.17	0.00
	C4	0.01	0.12	0.13	− 0.11	1.63	− 1.48	0.16	0.05
	C5	0.02	0.12	0.13	− 0.10	1.99	− 1.80	0.16	0.10
	C6	0.00	0.12	0.12	− 0.12	2.25	− 2.58	0.18	0.00
	C7	0.07	0.43	0.36	− 0.50	3.22	− 3.61	0.65	0.34

Units, mm, °.

**Figure 5:**
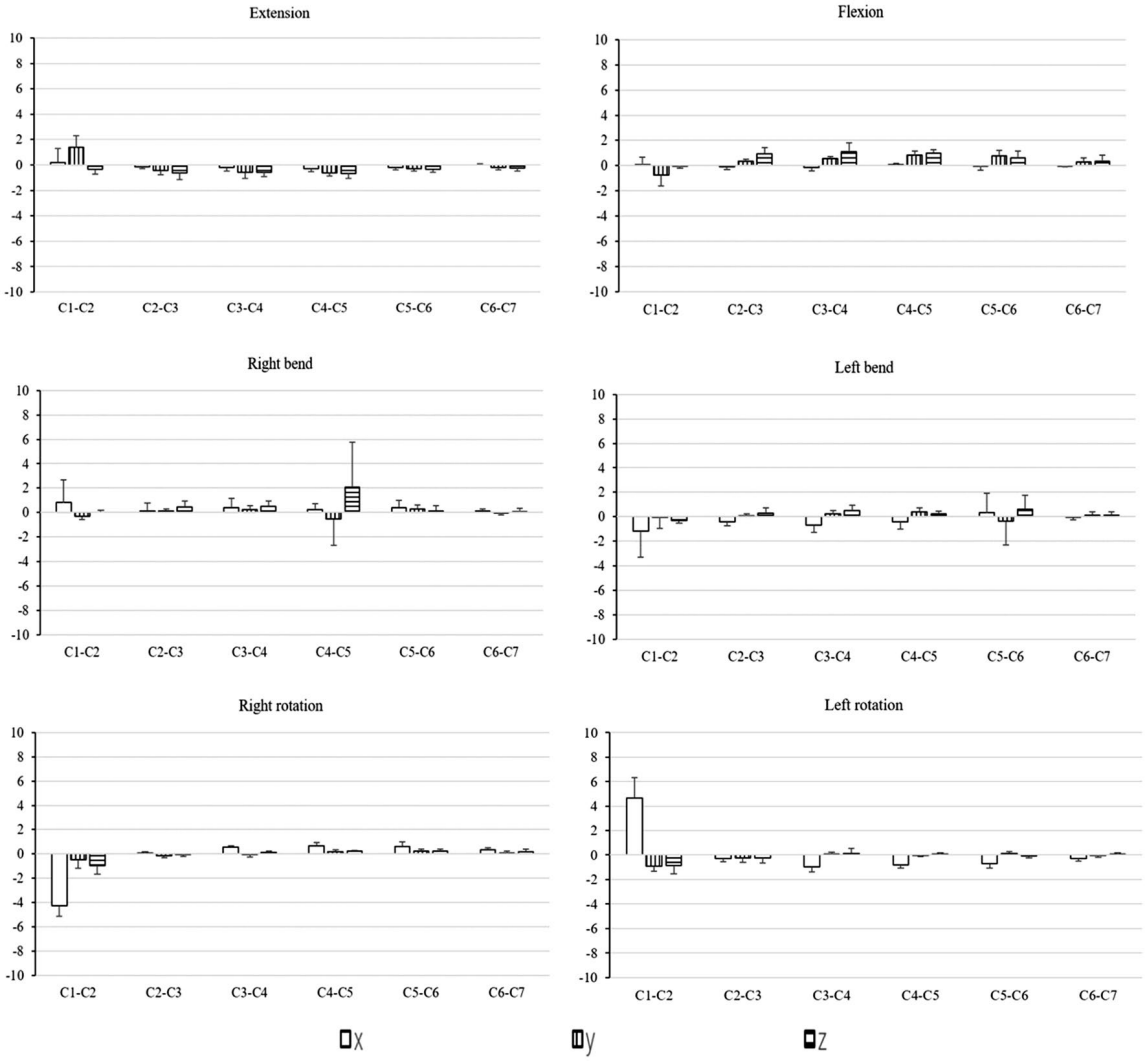
Translation (mm) changes of the cervical spine at six analyzed positions. x, right (−)-left ( +); y, inferior (−)-superior ( +); z, posterior (−)-Anterior ( +). A graphic legend indicating the meaning of the bar colors is shown in the bottom right of the figure.

**Figure 6:**
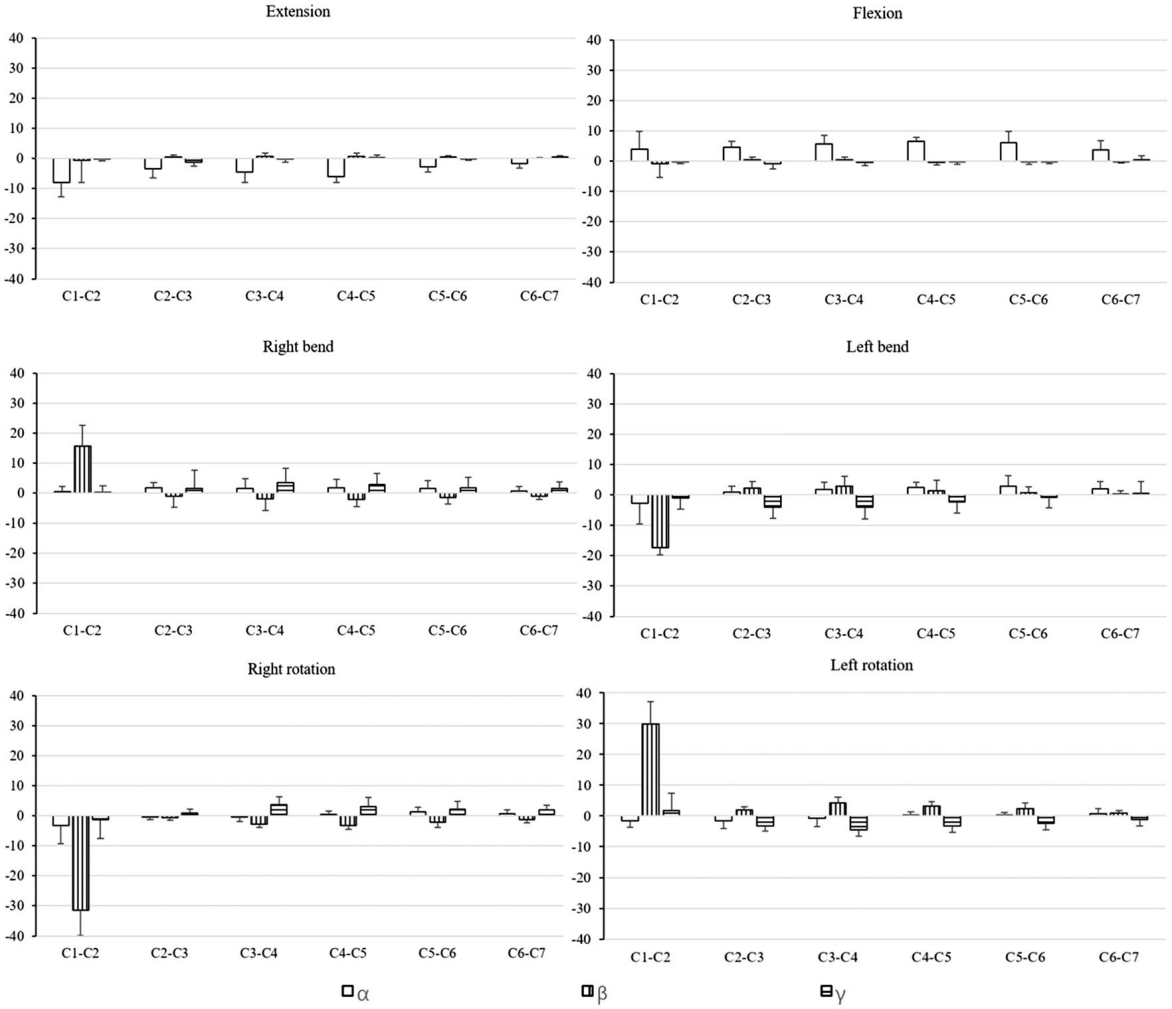
Rotation (°) changes of the cervical spine at six analyzed positions. Rotation (°): α, F (−)-E ( +); γ, LB left (−)-right ( +); β, AR right (−)-left ( +). A graphic legend indicating the meaning of the bar colors is shown in the bottom right of the figure.

Our in vivo cervical spine kinematic data are presented in Figs. 5 and 6. During head F–E, at the C1–C2 level, the primary flexion and primary extension extents were 4.03 ± 5.81° and 7.89 ± 4.78°, respectively. Translation in the three examined directions were generally < 1 mm, coupled with LB and AR that were < 1° (Table 2). During head LB, the extent of translation in the three ordinate directions were mostly < 1 mm and most of the movement occurred at the C1–C2 level, with mean displacements of 17.35 ± 2.38° leftward and 15.68 ± 7.03° rightward (Table 3). Head LB was coupled with AR in the direction opposite to the direction of LB at the C1–C2 level as well as minimal F–E motions at subaxial cervical levels. During the head AR assessment, we found that the mean primary AR extents at the C1–C2 level were 29.93 ± 7.19° for leftward rotation and 31.38 ± 8.49° for rightward rotation with some concomitant LB in the direction opposite to that of the AR coupled with extension (Table 4). At subaxial cervical levels, AR was coupled with LB in the same direction as the AR movement.

## Discussion

The present study introduces and provides validation of a combined CBCT imaging with 3D-3D registration technique for in vivo measurement of 6-DOF of cervical spine motions. We validated the accuracy of CBCT models relative to CT models, and then confirmed the accuracy of our CBCT data across six positions (F–E, AR left and right, and LB left and right), relative to neutral-position CBCT models. The present data show that the presently validated technique is feasible for analyses of the cervical spine over a wide range of motions.

We used a CT imaging reference model because CT images are known to be highly accurate^1,13,14,23^. Previously, Lim et al.^24^ used CT images of two cervical spines to show that a 3D spinal motion analysis method was accurate within 1 mm of translation and 1° of rotation. The accuracy of 3D surface models is dependent on several technical parameters, including detector sensitivity, x-ray beam inhomogeneity, and reconstruction technique limitations^25^. Spatial accuracy of CBCT specifically tends to be higher at the center of the volume than at the margins^26^. The present data indicate that mean deviations of CBCT imaged structures relative to CT images were in the range of 0.30–0.57 mm. For all six end positions of functional cervical spine movements evaluated in this study, the mean deviations relative to the neutral model were in the range of 0.14–0.67 mm and located mostly near the periphery of the vertebrae. The sites of maximum deviation were the tips of transverse processes, spinous processes, and the bony edges that form facet joints, which have little influence on overall accuracy in kinematic analyses. To augment our model registration accuracy, we used an automated surface-matching algorithm that employs point-based registration^27^. The main advantage of this method is that the entire 3D model surface is used for registration, rather than a predefined set of landmarks^28^. Although we obtained similarly accurate results with CT- and CBCT-based measurements, the CBCT method has several noteworthy benefits, including a low radiation dose (~ 2% of a regular neck CT exam) and an ability to provide clinically relevant information as well as novel 3D data for research^29^.

Previously, CT^1,14,15^, MRI^12^, and dual fluoroscopy^30–32^ techniques have been employed to obtain in vivo cervical spine measurements. Notably, Nagamoto et al.^12^ used MRI to compare cervical spine kinematics during head rotation between patients with cervical spondylopathy and an asymptomatic control group. However, MRI scans are time-intensive and MRI studies do not reflect the biomechanics of the spine under physiological loading because subjects are scanned in a supine position. Wang et al.^30^ investigated the ranges of motion of the three joints in the cervical spines (from C3 to C7) using biplane fluoroscopic imaging. However, due to skull occlusion, the general biplane technique has not reported the movement of the upper cervical spine. To the best of our knowledge, there are no previously published in vivo CBCT studies that investigated upper and subaxial cervical spine kinematics during F–E, LB, and AR of the head.

In agreement with prior studies^14,33^, the 6-DOF kinematic data reported herein indicate that intervertebral translational motions in ordinal directions are quite small during movements (mostly < 1 mm). Our data also show that most of the rotation of the head in AR occurs between C1 and C2 (31.66°), consistent with previous data^33,34^. This property can be attributed to the specialized anatomy of the upper cervical spine, including double convex joints in the lateral parts of C1 and C2 as well as the unique odontoid process (a.k.a. dens) on the C2 vertebra around which the C1 vertebra rotates, providing coupling mechanics that differ from those that occur at underlying levels. Upper cervical spine movements have complicated 3D mechanics consisting of both a main motion and coupled motions. In this study, we documented coupled LB at C1–C2 that was in the direction opposite to head F–E and AR movements. At subaxial cervical levels, we observed AR-coupled LB in the same direction as that of the AR. These patterns of motion are consistent with those first described by Panjabi et al.^35^ and confirmed in Ishii et al.’s^33^ 3D kinematic study.

Angular deviation analysis will greatly help to understand the findings in phase 2. As CBCT and CT scans were not performed simultaneously, it is not suitable to directly compare the angular deviation as the postures in two scans may be slightly different. Considering the geometric relationship between linear deviation and angular deviation, a proper estimation would be using the cervical spine dimension. The mean deviation (0.02–0.06 mm), taking into account a worst case over 11.9 mm cervical dimension (Table 5), will corresponding to 1/500 to 1/200 radius, which is approximately 0.1°to 0.3°. The proposed CBCT kinematic analysis can be used as an assisted diagnosis tool for patients without instrumentation. However, as a next step, it would also be a potential tool for the evaluation of surgery. We checked several post-op patients with instrumentation and found little metal artifact in CBCT scans (Appendix Fig. 1). In addition, similar mean absolute deviation of 0.2–0.3 mm were also found with respect to those without instrumentation (Appendix Fig. 2). In the future, with larger sample size, we will report kinematic analysis of post-op patients to investigate fixation and influence to adjacent levels.

**Table 5:** Typical 3D dimensions of cervical vertebrae.

	Height (mm)	Width (mm)	Length (mm)
C1	12.8	40.0	75.9
C2	37.4	43.8	56.8
C3	14.1	40.7	52.2
C4	13.5	36.9	47.0
C5	11.9	37.4	52.7
C6	11.9	49.8	56.5
C7	15.0	55.9	78.7

Several limitations of this study should be noted. Firstly, although CBCT radiation doses are lower than those of traditional CT, there is still some amount of radiation exposure. In future studies, protocols can be modified according to particular circumstances to further reduce radiation dose when possible, such as when spine motion does not need to be measured in all directions or by measuring upper or subaxial motions separately. Secondly, because our primary aim was to validate this technique with 10 subjects, only 5 additional healthy subjects were tested to verify clinical feasibility. We will pursue follow-up studies with additional healthy and pathological subjects. Third, the proposed method is suitable for patients with degenerative disease. Those with severe injury that cannot hold still may not took CBCT scans in seated positions. Nonetheless, the results of this study provide insights into the in vivo 3D mechanics of upper and subaxial cervical spine kinematics captured in human subjects enacting positions.

## Conclusion

The presently examined low-radiation CBCT modeling technique with 3D-3D registration was demonstrated to be valid for obtaining accurate measurements of cervical spine kinematics in vivo. The method yielded measurements of cervical spine vertebrae in six positions at the maximal extents of F–E, LB, and AR of the head with submillimeter deviations from reference models. Because this technique reproduces functional spine positions with 3D anatomic models, 6-DOF cervical spine kinematics can be obtained. The technique can be used for segmental kinematic analysis of the cervical spines, especially at the end positions of functional movements.

## Supplementary Information

Supplementary Information.
