# Supplementary material for: Validation and application of a novel in vivo cervical spine kinematics analysis technique
Source: Sci Rep. 2021 Dec 20;11:24266. doi: 10.1038/s41598-021-01319-x (PMC8688511; doi:10.1038/s41598-021-01319-x)
Supplement: Supplementary file 1 — Supplementary Information. [file 41598_2021_1319_MOESM1_ESM.pdf]

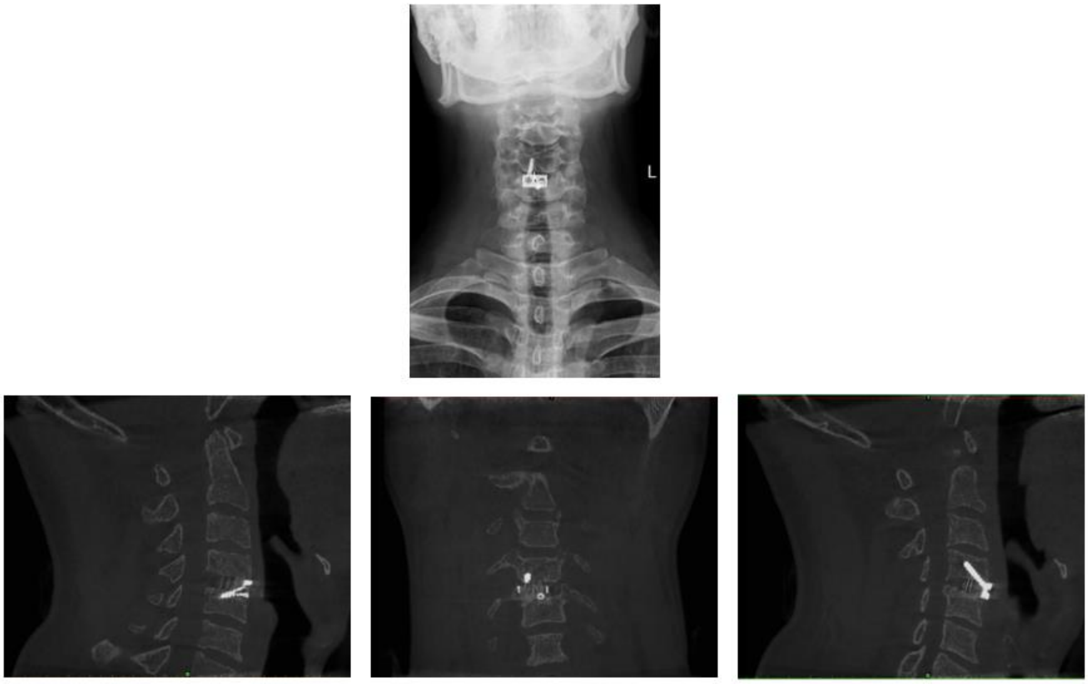

Appendix figure 1. Post-op patients with instrumentation and found little metal artifact in CBCT scans.

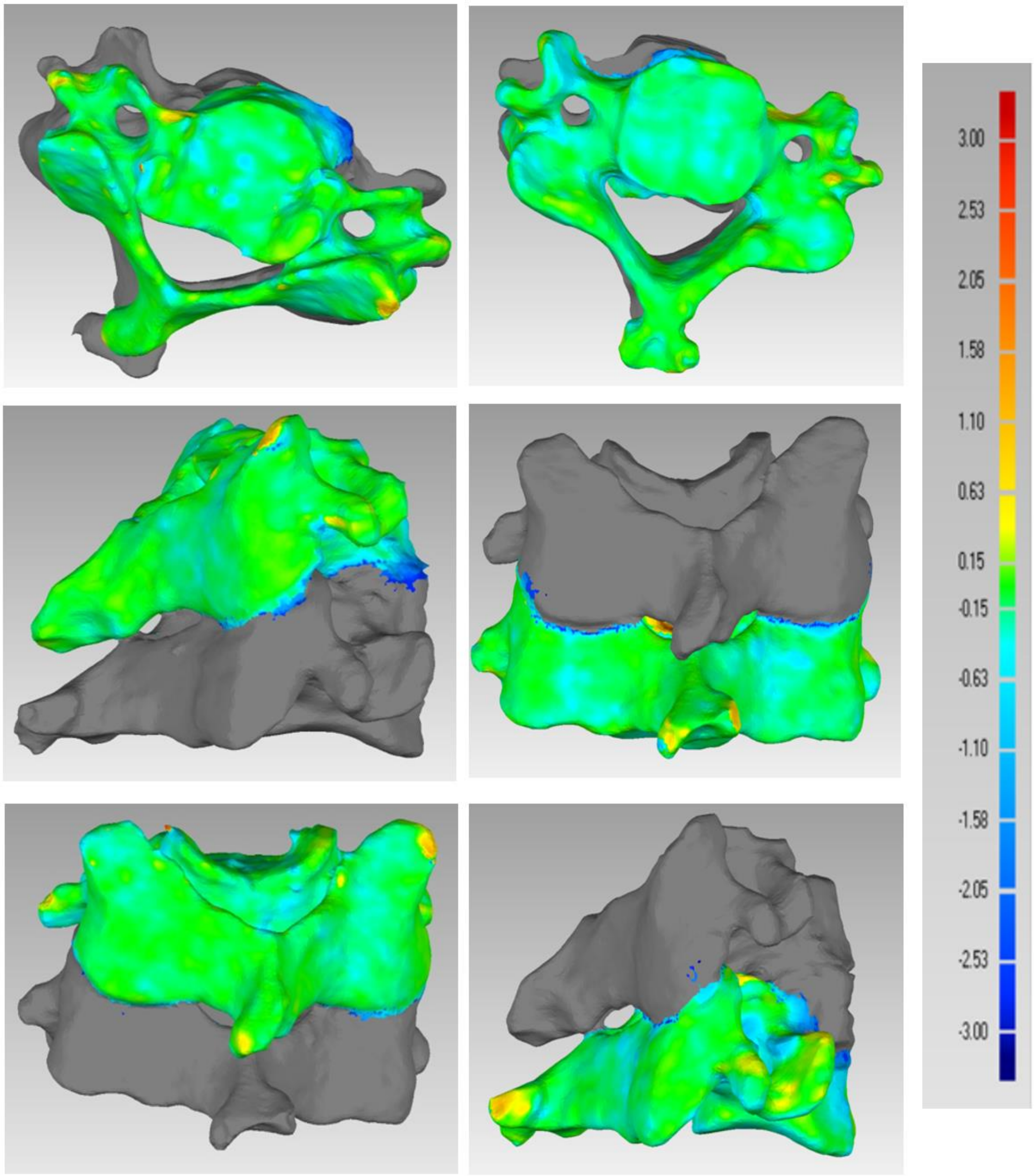

Appendix figure 2. Mean absolute deviation of 0.2-0.3 mm were also found with respect to those without instrumentation.
